# Editorial: White matter hyperintensities: the messages beneath and beyond

**DOI:** 10.3389/fnagi.2024.1367024

**Published:** 2024-01-19

**Authors:** Min-Chien Tu

**Affiliations:** ^1^Department of Neurology, Taichung Tzu Chi Hospital, Buddhist Tzu Chi Medical Foundation, Taichung, Taiwan; ^2^Department of Neurology, School of Medicine, Tzu Chi University, Hualien, Taiwan

**Keywords:** magnetic resonance imaging (MRI), white matter microstructural changes, white matter hyperintensities (WMH), white matter volume, segmentation (image processing), diffusion MRI, IVIM, coronary arterial lesions

White matter hyperintensity (WMH), an imaging feature that commonly exists in the aging spectrum, can negatively impact patients with variable neurological diseases. This clinical observation mainly stems from the fact that *micro-*vascular damage and white matter (WM) rarefaction are the principal pathologies corresponding to WMH formation (Schmidt et al., 2011). Since *micro*angiopathy can affect downstream neuronal integrity and alter the resistance of overall cerebral vascular trees (Schaeffer and Iadecola, 2021), exploring the biological significance beneath and beyond WMH gains neuroscientists' attention worldwide. Notwithstanding this, the delayed and latent property of WMH (Jiménez-Balado et al., 2019) makes detecting and monitoring WMH challenging. This addresses the unmet clinical need (Lawrence et al., 2015), in which identifying novel biomarkers with greater sensitivity that surrogate WMH-relevant neurobiological process is necessary.

Considering this, the researchers of this Research Topic entitled “*White Matter Hyperintensities—The Messages Beneath and Beyond*” by Frontiers in Aging Neuroscience endeavor to explore the biological interplay between WMH and the known/unknown factors. Using variable imaging biomarkers, we present the intricate associations between downstream and upstream changes relevant to WMH formation (Figure 1). The original articles cover variable populations, including vascular cognitive impairment (Lin et al.), cerebral small-vessel disease (Zhang et al.), and older subjects who are free from dementia (Pozo et al.; Jin et al.). The review articles focused on acute stroke (Wang et al.) and neurodegenerative diseases (Botz et al.), respectively.

**Figure 1:**
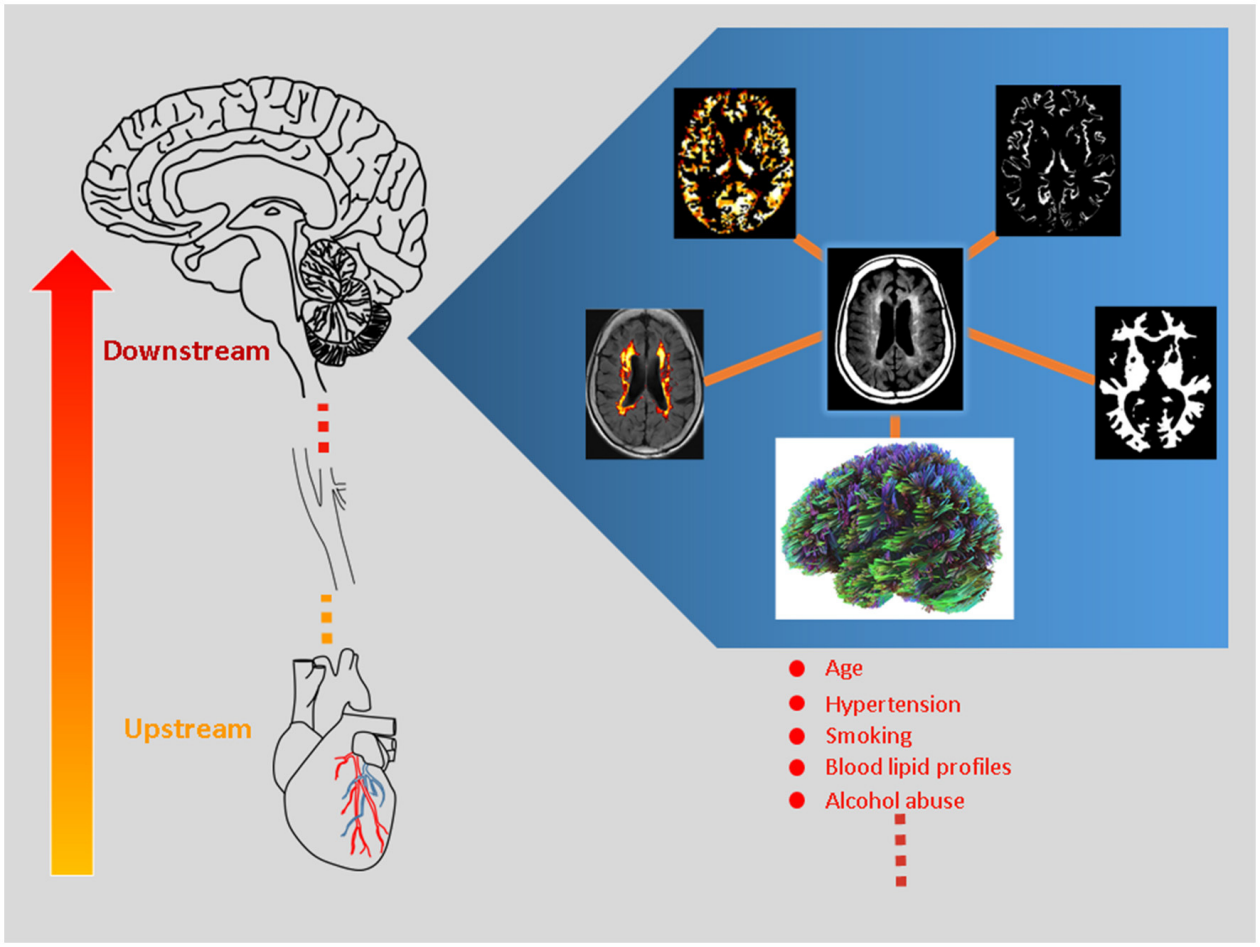
White matter hyperintensity (WMH) represents a proxy for *micro-*vascular injury, contributing to cerebral *micro*structural and *micro-*vascular perfusion changes (i.e., downstream pathogenesis). The figure illustrates the central concept of this Research Topic and highlights downstream neurobiological processes using state-of-the-art magnetic resonance imaging (MRI) techniques. The MRI method covers diffusion MRI, WMH volumetric algorithm, perfusion MRI, and segmentation analyses as clockwise illustrated. The clinical significance of these candidate imaging parameters is tested and reviewed. Moreover, the large-small artery interactions are explored within the brain-heart axis, where the coronary artery pathology is related to WHM progression (i.e., upstream mechanism).

Through the meta-analysis conducted by Wang et al., both periventricular and deep WMH are associated with hemorrhagic transformation among patients with ischemic stroke. Together with the spatial effect of WMH summarized by Botz et al., the significance of WMH topology likely varies by clinical phenotype/diagnosis. As such, the effect of WMH should be detailed in each disease entity alongside the general knowledge driven overall.

Zhang et al. reported the advantage of the volumetric assessment of WMH and WM over the Fazekas Scale in predicting cognitive impairment among patients with cerebral small-vessel disease. Consistent with their main findings, significant inverse associations between WMH and cognition were identified. Jin et al. highlighted a critical perspective regarding the brain-heart axis interactions. From their dedicated work, the coronary artery calcium score predicts WMH progression after a 12-month interval. Using intravoxel incoherent motion, Lin et al. explored parameter alterations within areas *involving* and *free* from WMH among patients with vascular cognitive impairment. Their study design shows that the parenchymal diffusivity is the most robust parameter that contrasts changes across tested regions. This aligns with the recent publication in which several candidate variables from diffusion metrics provide additional power than the original WMH effect in stratifying dementia patients (Tu et al., 2021). Pozo et al. reported that WMH within the frontal region acts as a mediator between frailty and executive dysfunction among older subjects who are still free from dementia.

The current Research Topic highlights several trends. Aside from well-known interactions among WMH, cognition, and frailty, the possibility of the interactions between large and small vessel diseases has been raised from clinical perspectives. From a radiological perspective, automatic segregation analysis can provide additional benefits regarding sensitivity to mirror subtle WMH changes by group or with time. This aids the conventional WMH measures that are semi-quantified through visual inspection. From a research perspective, state-of-the-art imaging metrics representative of WMH volume, WM *micro*structures, and *micro-*vascular perfusion are examined. These trials narrate the *in-vivo* observations of neurobiological processes relevant to WMH and pave the path for validating their use in clinical settings. There are still several challenges in the research field of WMH. First, the ceiling or flooring effects of the semi-quantitative measures for WMH (e.g., Fazekas scale) can exist. This is in line with the observed non-linear associations between WMH and clinical index (Wang et al.), hence highlighting the need for adjuvant WMH quantification measures with greater statistical power (Tubi et al., 2020). A proper automatic WMH quantification algorithm, preferably supervised by humans, would also extend our understanding of the clinical significance of WMH topology. As echoed by Botz et al., the methodology in characterizing the spatial distribution of WMH should consider the biological base and anatomical knowledge. Second, given that most studied populations are older subjects, a more sophisticated study design in the future would be warranted to disentangle the age and disease effects. Third, as imaging biomarkers in neurodegenerative diseases do not evolve in a parallel manner (Gong et al., 2017; Tu et al., 2022) or manifest as linear trajectory (Zamboni et al., 2019), exploring stage-dependent changes of each imaging biomarker can provide valuable information for its clinical application. There could be an additional benefit of integrating multimodal imaging metrics, where the results shall be carefully interpreted on considering the trade-off between metric dimension and neurobiological bases.

Overall, this Research Topic provides informative findings of imaging biomarkers that show their potential in WMH detection, WMH progression, and prognostic prediction. The messages delivered by these scientific works provide valuable references to optimize the clinical workflow and diagnostic repertoire.

## Author contributions

M-CT: Writing – original draft, Writing – review & editing.
